# Mouse promyelocytic leukemia zinc finger protein (PLZF) regulates hepatic lipid and glucose homeostasis dependent on SIRT1

**DOI:** 10.3389/fphar.2022.1039726

**Published:** 2022-11-10

**Authors:** Huiling Hu, Nannan Sun, Haiyan Du, Yuqing He, Kunyi Pan, Xiuli Liu, Xiaoxia Lu, Jie Wei, Mianmian Liao, Chaohui Duan

**Affiliations:** ^1^ Department of Clinical Laboratory, Sun Yat-Sen Memorial Hospital, Sun Yat-Sen University, Guangzhou, China; ^2^ Guangdong Provincial Key Laboratory of Malignant Tumor Epigenetics and Gene Regulation, Sun Yat-Sen Memorial Hospital, Sun Yat-Sen University, Guangzhou, China; ^3^ Department of Obstetrics and Gynecology, Guangdong Provincial Key Laboratory of Major Obstetric Diseases, The Third Affiliated Hospital of Guangzhou Medical University, Guangzhou Medical University, Guangzhou, China; ^4^ State Key Laboratory of Ophthalmology, Zhongshan Ophthalmic Center, Sun Yat-sen University, Guangzhou, China; ^5^ Department of Hepatology, Shenzhen Traditional Chinese Medicine Hospital, Shenzhen, China; ^6^ Translational Medicine Research Center, Zhujiang Hospital, Southern Medical University, Guangzhou, China

**Keywords:** PLZF, SREBP-1c, SIRT1, lipid homeostasis, glucose homeostasis

## Abstract

Previous studies have demonstrated that promyelocytic leukemia zinc finger protein (PLZF) promotes the expression of gluconeogenic genes and hepatic glucose output, which leads to hyperglycemia. However, the role played by PLZF in regulating lipid metabolism is not known. In this study, we aimed to examine the function of PLZF in regulating hepatic lipid and glucose homeostasis and the underlying mechanisms. The expression of PLZF was determined in different mouse models with regard to non-alcoholic fatty liver disease (NAFLD). In the next step, adenoviruses that express PLZF (Ad-PLZF) or PLZF-specific shRNA (Ad-shPLZF) were utilized to alter PLZF expression in mouse livers and in primary hepatocytes. For the phenotype of the fatty liver, histologic and biochemical analyses of hepatic triglyceride (TG), serum TG and cholesterol levels were carried out. The underlying molecular mechanism for the regulation of lipid metabolism by PLZF was further explored using luciferase reporter gene assay and ChIP analysis. The results demonstrated that PLZF expression was upregulated in livers derived from ob/ob, db/db and diet-induced obesity (DIO) mice. Liver PLZF-overexpressing C57BL/6J mice showed fatty liver phenotype, liver inflammation, impaired glucose tolerance and insulin sensitivity. On the other hand, hepatic PLZF knockdown in db/db and DIO mice alleviated hepatic steatosis. Of note, we found that PLZF activates SREBP-1c gene transcription through binding directly to the promoter fragment of this gene, which would induce a repressor-to-activator conversion depending on its interaction with SIRT1 in the role played by PLZF in the transcription process through deacetylation. Thus, PLZF is identified as an essential regulator of hepatic lipid and glucose metabolism, where the modulation of its liver expression could open up a therapeutic path for treating NAFLD.

## Introduction

Hepatic lipogenesis dysfunction accelerates the pathogenesis of metabolic diseases, including non-alcoholic fatty liver disease (NAFLD). In NAFLD, fatty acids are ectopically accumulated in the liver (Browning and Horton, 2004). NAFLD, which is defined as a spectrum of hepatic disease ranging from hepatic steatosis to hepatic steatohepatitis, fibrosis, and cirrhosis, has expanded as a global threat, particularly in developed countries (Bedossa, 2017). Although the pathogenesis behind NAFLD is complicated, the activity modulation of a number of transcription factors responsible for regulating hepatic lipid and glucose homeostasis appears to be essential during the treatment of NAFLD. As an example of important transcription factors that take part in regulating hepatic lipid metabolism, researchers can point out the sterol regulatory element binding proteins (SREBPs) (Ahmed and Byrne, 2007).

Hepatic SREBP-1c is characterized as the primary member of the SREBP family, which constitutes major transcription factors responsible for the regulation of genes concerning cholesterol and fatty acid synthesis (Shimomura et al., 1997; Eberlé et al., 2004). Prior studies have revealed that multiple modulators regulate the expression of SREBP-1c. For example, KLF11 and Dec1 ameliorate hepatic steatosis *via* inhibition of SREBP-1c expression (Zhang et al., 2013; Shen et al., 2014). Thus, establishing the regulatory factors of SREBP-1c is crucial, because it provides novel insight into the potential therapeutic targets for the treatment of NAFLD.

The promyelocytic leukemia zinc finger (PLZF), also called ZBTB16 (zinc finger and BTB domain containing 16), is considered a transcription factor that takes part in regulating a number of different biological processes, such as spermatogenesis (Ching et al., 2010), stem cell maintenance (Liu et al., 2016), immune regulation (Xu et al., 2009) and invariant natural killer T cell (iNKT) development (Kovalovsky et al., 2008). Previous research uncovers the potential role played by PLZF in the pathogenesis of metabolic diseases. PLZF is an iNKT cells specific transcription factor that is necessary for its full functionality (Kovalovsky et al., 2008). In addition, PPARγ2 and PLZF synergically promote SREBP-1c transcription to increase lipid biosynthesis in iNKT cells (Fu et al., 2020). SNP (783C>G) in the PLZF coding sequence leads to nonsynonymous amino acid substitution–serine to threonine at position 208 (T208S)–which affects total body weight, adiposity, and the insulin sensitivity of the skeletal muscles (Seda et al., 2005). Furthermore, a study conducted by Liska et al. demonstrated that PLZF deficiency ameliorates metabolic and cardiac traits in the spontaneously hypertensive rat (Liška et al., 2017). Although a previous study has revealed that liver-overexpressed PLZF damaged glucose tolerance and insulin sensitivity *via* promoting the expression of the gluconeogenic gene and hepatic glucose output (Chen et al., 2014), the role played by PLZF in regulating lipid metabolism, including lipid synthesis, liver inflammation, and fatty acid oxidation, is still unknown.

Given what was stated above, we set out a goal to examine whether PLZF is involved in the regulation of hepatic lipogenesis *via* mediating the SREBP-1c expression. Our study demonstrated that PLZF has a crucial role in the regulation of lipogenesis. This process was dependent on its interaction with SIRT1, which allowed for a repressor-to-activator conversion in the role played by PLZF in the transcription process through deacetylation. Thus, PLZF is found to be a vital regulator of hepatic lipid metabolism, where the modulation of its liver expression may open up a therapeutic path for treating NAFLD.

## Materials and methods

### Animals

The experimental procedures concerning animals received approval from the Animal Care and Use Committee of Zhongshan Ophthalmic Center of Sun Yat-sen University; they were carried out in accordance with all relevant ethical regulations. Male ob/ob, db/db, db/m, and C57BL/6J mice (aged six to 8 weeks) were acquired from the Model Animal Research Center of Nanjing University (Nanjing, China) and were kept in standard cages. The cages were placed in a specific pathogen-free facility, in which a 12-h light/dark cycle was maintained. The animals were subjected to free access to food and water. Liver-specific SIRT1-knockout (SIRT1^−/−^) mice featuring conditional delete of SIRT1 exon four were the result of crossing between mice with floxed alleles of SIRT1 and liver Cre recombinase-expressing mice (Cheng et al., 2003). For the DIO model and the normal diet model used as control, mice were fed *ad libitum* with either a normal chow (10% fat, Lab Diet) or high-fat diet (60% fat, Research Diets) with free access to water. For adenovirus injection, mice were injected *via* their tail vein with adenovirus expressing 1) green fluorescent protein (Ad-GFP), 2) PLZF (Ad-PLZF), and 3) short-hairpin (sh) RNA against luciferase (Ad-shCON) or shRNA against PLZF (Ad-shPLZF) as controls (0.5–1.0 × 10^9^ pfu/mouse in 150 μl PBS). In the next step, after 5–7 days elapsed from the infection, the liver and plasma of the mice were retrieved for further analysis. The grouping of all the mice was performed in a random manner, and the researchers performing the experiments were blind to the assignment of the groups and the evaluation of the outcome.

### Preparation of expression plasmids and recombinant adenoviruses

The full-length mouse PLZF and SIRT1 gene were amplified from liver cDNA of C57BL/6J mice. Afterward, Myc-tagged PLZF and Flag-tagged SIRT1 were cloned into pcDNA3.1 utilizing the following PCR primers: 5′-CCG​GGT​ACC​ATG​GAT​CTG​ACA​AAG​ATG​GGG​AT-3’ (Forward) and 5′-CCG​G​AT​ATC​CAG​ATC​CTC​TTC​AGA​GAT​GAG​TTT​CTG​CTC​CAC​ATA​ACA​CAG​GTA​GAG​GTA​CGT-3’ (Reverse) for PLZF or 5′-CCG​GGT​ACC​ATG​GCG​GAC​GAG​GTG​GCG​CT-3’ (Forward) and 5′-CCG​CTC​GAG​GCT​TAT​CGT​CGT​CAT​CCT​TGT​AAT​CTG​ATT​TGT​CTG​ATG​GAT​AGT-3’ (Reverse) for SIRT1. PLZF- or SIRT1-expressing recombinant adenoviruses were generated following what was described in ref. (Luo et al., 2007). PCR was used for the amplification of the mouse SREBP-1c gene promoter (-500 to -1 bp) utilizing mouse genomic DNA. Then, SREBP-1c gene promoter was inserted into a pGL3-basic luciferase reporter vector (p-SREBP-1c-500). A series of 5′ truncated constructs of the SREBP-1c gene promoter (p-SREBP-1c-224, p-SREBP-1c-119, p-SREBP-1c-71) were prepared by PCR using p-SREBP-1c-500 as a template. The primer sequences used are listed in Supplementary Table S1.

### RNA interference

The design and synthesis of shRNAs targeting PLZF gene (shPLZF), SIRT1 (shSIRT1), or luciferase (shCON) were both carried out using the shRNA design program of Genepharma website (Genepharma, Shanghai, China). The shRNAs were constructed into adenovirus plasmids, where the adenoviruses were generated based on procedures given in the reference (Luo et al., 2007). The sequences of the shRNAs were shown as follows: shPLZF: TGG​AAA​TGA​TGC​AGG​TAG​A, shSIRT1: GCA​CCG​ATC​CTC​GAA​CAA​TTC and shCON:5′-CTTACGCTGAGTACTTCGA-3’.

### Isolation of mouse primary hepatocytes and their culture

The isolation of mouse primary hepatocytes and their culture was done following reference (Zhang et al., 2013). In brief, type II collagenase (0.5 mg/ml) perfusion *via* the inferior vena cava was administered for the isolation of primary hepatocytes from C57BL/6J and Ko-SIRT1 mice. The viability assessment of hepatocytes was carried out using the trypan blue exclusion method. For the experiments, cells with viability >95% were used. For the culture of mouse hepatocytes, RPMI-1640, which contained FBS (10%), penicillin (with concentration of 100 units/ml), and streptomycin (with concentration of 0.1 mg/ml), was used. In general, for overexpression experiments, we used 100 multiplicity of infection (MOI), and for shRNA knockdown experiments six00 MOI. Afterward, the harvesting of the cells was carried out after elapse of 1–2 days from the infection.

### Analytical procedures and chemicals

A commercial kit (Jiancheng, Nanjing, China) was used for the purpose of determining the serum concentrations of TG and cholesterol following the manufacturer’s instructions. For determining the intracellular TG and cholesterol content, first cells were lysed with Triton X-100 (0.1%) and then a commercial kit (Jiancheng, Nanjing, China) was employed for assessing the TG and cholesterol content of cell lysates. Hepatic TG and cholesterol were extracted with chloroform-methanol (2:1) according to reference. (Folch et al., 1957). and then their concentrations were measured by a commercial kit (Jiancheng, Nanjing, China). The concentrations of serum TNFα and IL-6 were measured using ELISA (R&D Systems, Minneapolis, United States). EX-52 and Resveratrol were purchased from MedChemExpress.

### Real-time qRT-PCR analysis

The isolation of the RNA present in the mouse primary hepatocytes or fresh liver tissues was conducted by means of a TRIzol-based method (Invitrogen). Reverse transcription was performed according to the instructions of the manufacturer of the 5× All-In-One RT MasterMix kit (Applied Biological Materials Inc., Richmond, Canada). qRT-PCR was carried out utilizing SYBR Green Master mix with LightCycler^®^ 96 Real-Time PCR System (Roche). Three independent biological replicates were carried out, and the genes’ relative expression levels were calculated with the 2^−ΔΔCT^ method. The data of the gene expression were normalized to the expression levels of β-actin. The sequences of the primer can be found in Supplementary Table S2.

### Immunoprecipitation

For adenoviral transductions, HEK293T cells were transduced with adenovirus expressing Myc-tagged PLZF or Flag-tagged SIRT1. Once 36 h elapsed from transduction, whole cell lysates were retrieved by centrifuging (12,000 rpm; 10 min; 4°C). An amount of 1–1.2 mg of the lysates was utilized for the immunoprecipitation test. The incubation of lysates was carried out with anti-Flag antibody or anti-Myc antibody overnight (4 °C). Then, a 2 h treatment with protein A/G Sepharose was performed. After three-time rinsing with PBS, the process of retrieving the immunoprecipitated proteins from the beads was carried out through a 10 min boiling in sample buffer or by competition with the tag peptide. Afterward, immunoblotting was used for the analysis of immunoprecipitated proteins. Anti-FLAG mouse mAb (8146; CST) and Myc mouse mAb (2276; CST) were used against epitope tags.

### Western blotting

The lysis of cells and tissues was carried out in RIPA lysis buffer [Tris-HCl (with concentration of 50 mM, pH 8), NaCl (with concentration of 150 mM), Triton (1%), SDS (with concentration of 0.1%), EDTA (with concentration of 1 mM), and deoxycholic acid (with concentration of 0.5%)] plus a cocktail containing protease and phosphatase inhibitors (Roche), and another one comprising deacetylase inhibitors (Santa Cruz) for 30 min at 4°C. SDS-PAGE was used for the separation of an amount of 30–50 μg of proteins, which was transferred to PVDF membranes (Merck Millipore). Overnight incubation (4°C) with antibodies was applied to the membranes; the antibodies were the following: anti-SREBP-1 (sc-13551, Santa Cruz), anti-SIRT1 (13161-1-AP, Proteintech), PLZF (sc-28319, Santa Cruz), anti-FAS (3180, CST), anti-acetyl Lysine antibody (9441, CST) and β-Actin (bs-0061R, Bioss). For ac-PLZF examination, immunoprecipitation with an anti-PLZF antibody was introduced into cells and tissues extracts, which was followed by immunoblotting with an anti-acetyl-lysine antibody (α-AcK), so that only the acetylated PLZF protein was detected (McConnell et al., 2015; Sadler et al., 2015). Detection of protein signals was carried out by incubation with horseradish peroxidase-conjugated secondary antibodies, where ECL detection reagent (Pierce) was employed in the next step following the manufacturer’s instructions.

### Histological and immunohistochemistry analyses

For H&E staining, neutral-buffered formalin (10%) was used for fixing the liver tissues; afterward, the tissues were paraffin-embedded, followed by being cut into sections (thickness = 7 μm) Liquid nitrogen was used for freezing the liver tissues for Oil red O staining; the frozen tissues were then cut into sections with a thickness of 10 µm. After staining the sections, their analysis was carried out at magnification of ×200 utilizing a Zeiss Axio Observer microscope.

### Analysis of glucose output assay

Primary mouse hepatocytes were seeded into six-well plates. In the next step, the indicated adenovirus was used for infecting them. Once 36 h elapsed from infection, the cells were washed with PBS (three times); afterward, they were incubated in 1 ml/well of phenol-red-free, glucose-free DMEM that contained dexamethasone (1 μM), pyruvate (2 mM), lactate (20 mM) and forskolin (10 μM) for 3–6 h. An Amplex Red Glucose/Glucose Oxidase Assay Kit (Applygen Technologies, Beijing, China) was used for determining the concentration of glucose in the medium. The lysis of the cells was performed, and for each lysate, the protein concentration was measured (Bio-Rad, Hercules, CA, United States). The normalization of the glucose output rate was carried out using the cellular protein content.

### Analysis of luciferase reporter gene assay

The co-transfection of mouse SREBP-1c promoter constructs (PGL3-SREBP-1c-500, PGL3-SREBP-1c-224, PGL3-SREBP-1c-119, and PGL3-SREBP-1c-71) with PLZF-expressing plasmid (pcDNA3.1-PLZF) or empty vectors (pcDNA3.1) was carried out into HepG2 cells utilizing Lipofectamine™ 2000 (Invitrogen), in accordance with the instructions of the manufacturer. As an internal control, a vector expressing Renilla luciferase was utilized. After elapse of 48 h, the harvesting of the cells was performed to evaluate the luciferase activity utilizing the Dual-Luciferase reporter assay system (Promega), following the manufacturer’s instruction.

### Analysis of chromatin immunoprecipitation assay

ChIP assays were carried out using the procedure of a previous report (Sun et al., 2021). In brief, the crosslinking of the tissues was performed in formaldehyde (1%) at 37°C (15 min), followed by resuspension in 200 ml of lysis buffer [Tris-HCl (with concentration of 50 mM; pH 8.1), SDS (with concentration of 1%), and EDTA (with concentration of 10 mM)]. Lysates were sonicated; afterward, they were diluted using CHIP dilution buffer [SDS (with concentration of 0.01%), Triton X-100 (with concentration of 1.1%), Tris-HCl (with concentration of 16.7 mM; pH 8.1), NaCl (with concentration of 167 mM), and EDTA (with concentration of 1.2 mM)]. The diluted lysates were subjected to immunoprecipitation utilizing anti-PLZF antibody or normal mouse IgG. afterward, the immunoprecipitates were washed followed by elution with 300 ml of elution buffer [NaHCO_3_ (with concentration of 0.1 M) and SDS (with concentration of 1%)] and reversed. The amplification of promoter region of SREBP-1c was carried out by PCR utilizing these primers: 5′-GAT​TGG​CCA​TGT​GCG​CTC​A-3′ as a forward primer and 5′-CCT​TCA​AAT​GTG​CAA​TCC​ATG -3′ as a reverse primer.

### Statistical analysis

Data shown were presented as mean ± standard error of the mean (SEM). The software package IBM SPSS statistics (version 22.0) was employed for performing the statistical analysis. One-way ANOVA was utilized for the purpose of evaluating differences among groups followed by Bonferroni’s post hoc test. For making comparisons between two groups, differences were assessed by Student’s t-test. *p* < 0.05 was set as statistically significant.

## Results

### Hepatic PLZF expression was increased in hepatic steatosis

**FIGURE 1:**
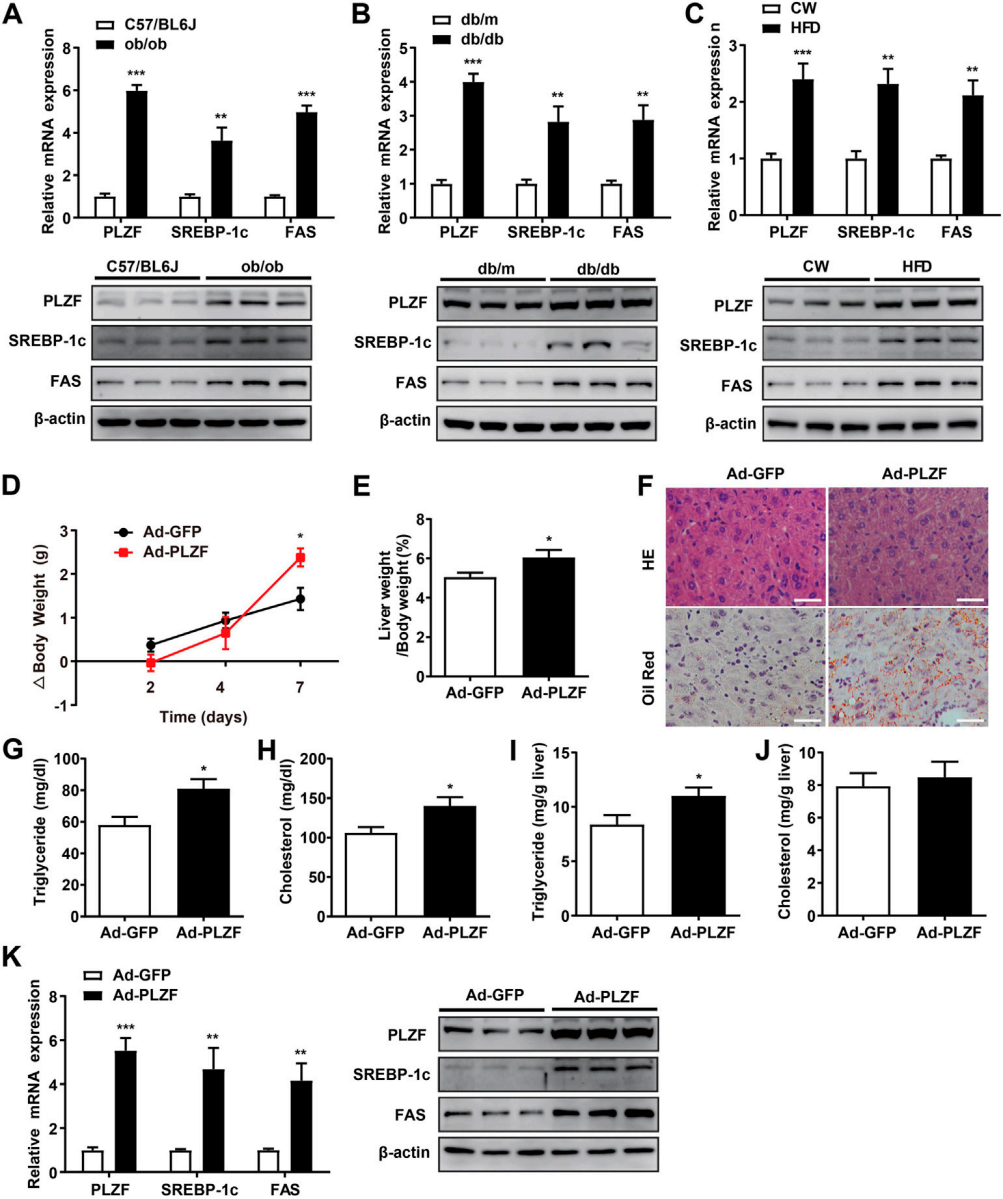
Hepatic PLZF gene expression is upregulated in NAFLD mice and adenovirus-mediated PLZF overexpression in C57BL/6J mouse livers leads to improvement in the fatty liver phenotype. PLZF, SREBP-1c, and Fas expression is presented in qPCR analysis (top panel) and western blot analysis (bottom panel). **(A)** C57BL/6J control mice and ob/ob mice, **(B)** db/m control mice and db/db mice, and **(C)** C57BL/6J mice that were fed a normal chow or a high-fat diet for 16 weeks (DIO mice). Ad-GFP or Ad-PLZF adenovirus was injected into C57BL/6J mice (male). Mice were sacrificed for further analysis. Depicted are **(D)** body weight change, **(E)** liver weight to body weight ratio, **(F)** representative section staining of livers with H&E (top panel) and Oil Red O (bottom panel), **(G)** serum TG, **(H)** serum cholesterol, **(I)** hepatic TG, **(J)** hepatic cholesterol. SREBP-1c and Fas in livers of mice treated with the indicated adenovirus were analyzed with **(K)** qPCR analysis (left panel) and western blot analysis (right panel). N = 6–8/group. For adenovirus injection, mice were injected *via* their tail vein with adenovirus. Then, 5–7 days after infection, mice livers and plasma were collected for further analysis. The data shown are the means ± SEM. Scale bar, 50 μm *p < 0.05, **p < 0.01, ***p < 0.001.

Previous reports indicate that hepatic PLZF expression is upregulated depending on the time course of fasting and stimulation of hepatic gluconeogenesis (Chen et al., 2014). However, whether and how PLZF plays role in hepatic lipogenesis are still unclear. This prompted us to examine its role in hepatic lipid metabolism. Three different NAFLD mouse models, namely ob/ob, db/db, and DIO mice, were used to detect hepatic expression of PLZF by qPCR and western blot analysis. The results demonstrate that hepatic PLZF expression is dramatically increased in NAFLD mice in comparison to the control mice, where a significant elevation of lipogenic genes, including SREBP-1c and fatty acid synthase (FAS) was observed (Figures 1A–C). In line with this findings, 75 μmol/L palmitic acid treatment, which mimics the high-fat stress to establish an *in vitro* model of lipid accumulation, significantly upregulated PLZF expression in primary mouse hepatocytes (Supplementary Figure S1A). To further study the contributions of PLZF to hepatic steatosis, adenovirus expressing Myc-tagged PLZF (Ad-PLZF) was generated and was injected *via* tail vein into wild-type mice with standard chow diet, which resulted in a specifically increased hepatic PLZF overexpression. Although no change was observed in the food intake (Supplementary Figure S1B), hepatic PLZF overexpression induced significant increases in the body weight and the value of the ratio of liver weight/body weight (Figures 1D,E). Furthermore, our histologic analysis (including Oil red O and HE stainings) demonstrated that Ad-PLZF treatment apparently stimulated the hepatosteatosis, including inducing massive deposit of large lipid droplets and ballooning degeneration of liver cells, which marked a process that leads to NAFLD and finally to non-alcoholic steatohepatitis (NASH) (Figure 1F). In line with histological analysis, biochemical analysis showed that adenovirus-mediated overexpression of PLZF dramatically increased the hepatic TG and serum TG and cholesterol levels (Figures 1G–J). We also examined the role of PLZF overexpression on hepatic lipogenesis genes. Similar to the ob/ob, db/db, and DIO mice, hepatic PLZF overexpression robustly induced an increase in SREBP-1c and Fas gene expression in liver (Figure 1K), which could lead to hepatic steatosis.

### Silencing of hepatic PLZF protects db/db mice against hepatic steatosis

**FIGURE 2:**
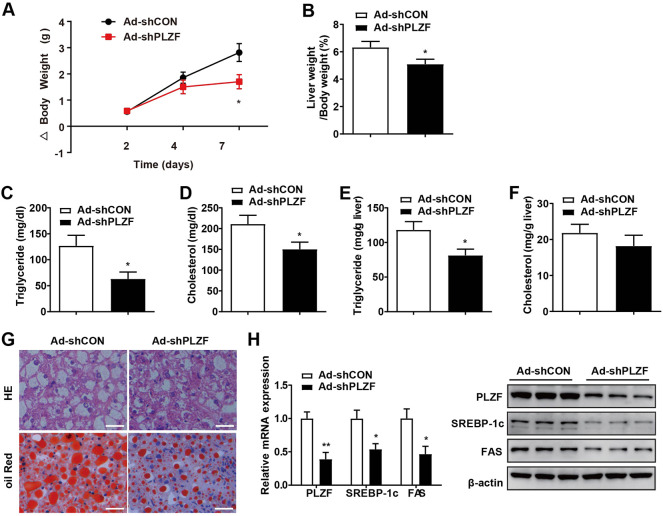
Knockdown of hepatic PLZF in db/db mice induced improvement in the fatty liver phenotype. Db/db mice were injected with Ad-shCON or Ad-shPLZF adenovirus and then were for further analysis. Depicted are **(A)** body weight change, **(B)** liver weight to body weight ratio, **(C)** serum TG, **(D)** serum cholesterol, **(E)** hepatic TG, **(F)** hepatic cholesterol, **(G)** liver sections stained with H and E (top panel) and Oil Red O (bottom panel). SREBP-1c and Fas in livers of mice that were treated with the indicated adenovirus were analyzed by **(H)** qPCR analysis (left panel) and western blot analysis (right panel). N = 6–8/group. For adenovirus injection, mice were injected *via* their tail vein with adenovirus. Then, 5–7 days after infection, mice livers and plasma were collected for further analysis. The data shown are the means ± SEM. Scale bar, 50 μm *p < 0.05, **p < 0.01.

To further comprehend the pathogenic role of hepatic PLZF in NAFLD progression, we produced an adenovirus that expressed PLZF-specific shRNA (Ad-shPLZF) and injected Ad-shPLZF into NAFLD models, db/db mice with chow diet *via* tail vein. As expected, hepatic PLZF knockdown slightly, but significantly, slowed body weight gain, where Ad-shPLZF infection decreased the liver weight (Figures 2A,B). Moreover, knockdown of PLZF in the liver showed an obvious reduction in both hepatic and serum TG content, where the serum cholesterol level also decreased (Figures 2C–F). Thus, in db/db mice, hepatic-specific knockdown of PLZF visibly alleviated the phenotype of the fatty liver, as there was reduction in large lipid droplets and ballooning degeneration of liver cells (Figure 2G). Importantly, similar results were also obtained for DIO mice (Supplementary Figure S2), except for body weight gain was not significantly changed. To further evaluate the underlying molecular mechanism in place, we performed *in vitro* assay tests, where we found that consistent with the phenotype, the lipogenic genes’ expression, including SREBP-1c and Fas, was dramatically suppressed by the knockdown of PLZF in the liver tissues obtained from db/db and DIO mice (Figure 2H and Supplementary Figure S2H), which contributed to the attenuation of NAFLD phenotype.

### Hepatic PLZF enhances SREBP-1c expression *via* binding its promoter

**FIGURE 3:**
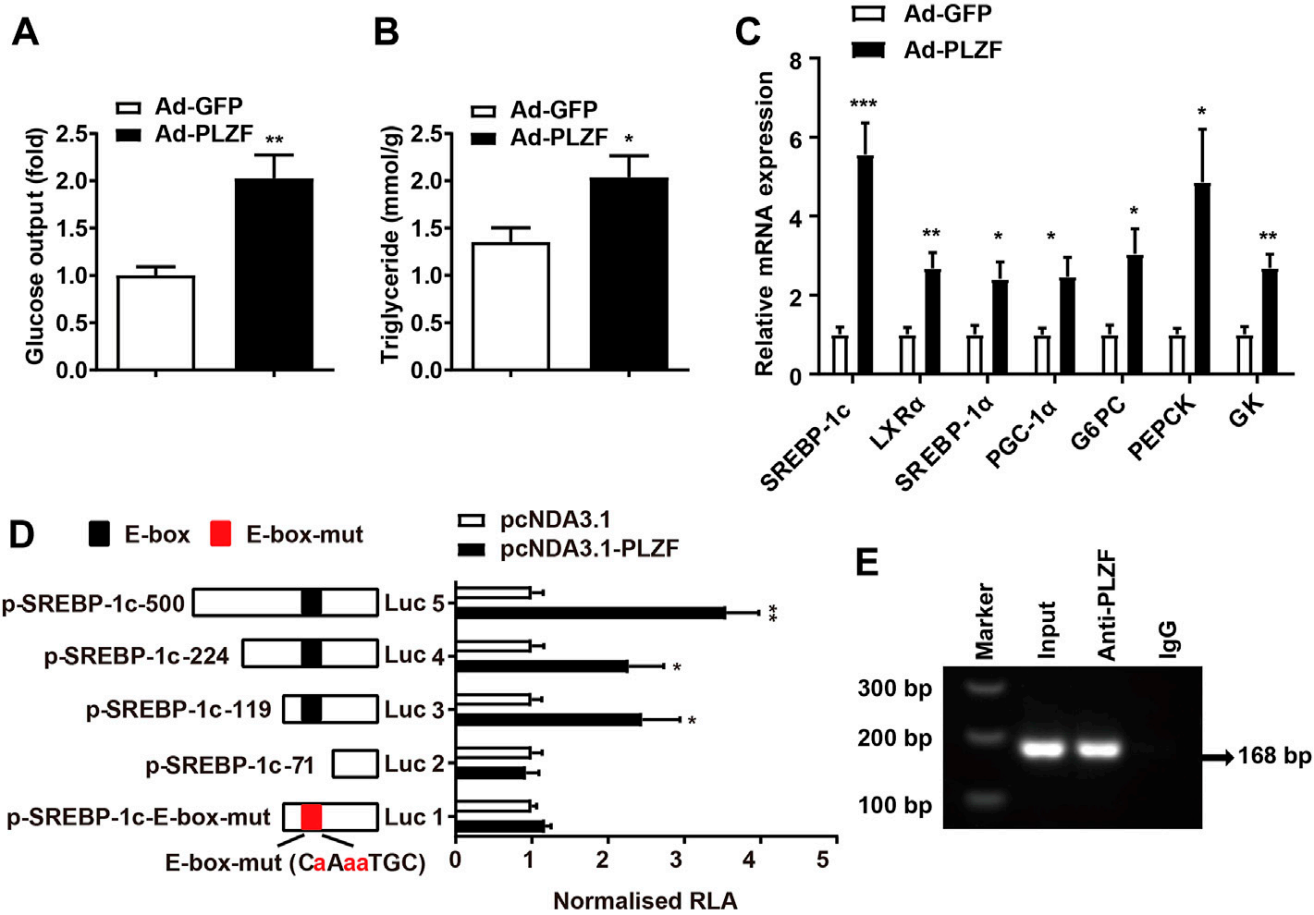
PLZF stimulates transcription of the SREBP-1c gene by directly binding to its promoter. **(A)** Glucose production and **(B)** TG levels in the primary hepatocytes treated with the indicated adenovirus. **(C)** qPCR analysis of glucogenic genes and lipogenic genes in the primary hepatocytes treated with the indicated adenovirus. **(D)** Luciferase reporter gene assay in HepG2 cells transfected with the indicated plasmids. A number of truncated SREBP-1c promoters that were fused to the luciferase reporter gene were co-transfected with pcDNA3.1 or pcDNA3.1-PLZF plasmid into HepG2 cells. **(E)** For the purpose of assessing endogenous PLZF occupancy of the SREBP-1c promoter, ChIP analysis was performed on liver tissues isolated from C57BL/6J mice. N = 4–6/group. The data shown are the means ± SEM. *p < 0.05, **p < 0.01, ***p < 0.001. Luc, luciferase. RLA, relative luciferase activity.

To further delineate the mechanism of PLZF-induced hepatic steatosis, we performed *in vitro* experiments. According to the *in vitro* tests, consistent with previous reports (Chen et al., 2014), primary hepatocytes with Ad-PLZF transfection showed a robust increase in glucose production, where the TG levels contents were also dramatically increased (Figures 3A,B). Subsequent qPCR analysis also indicated that overexpression of PLZF in primary hepatocytes upregulated both glucogenic genes and lipogenic genes, especially SREBP-1c and Liver X receptor *a* (LXRα) (Figure 3C). SREBP-1c is regulated by multiple molecular pathways and has been found to be a predominant transcription factor playing a part in lipid homeostasis in liver (Eberlé et al., 2004). To determine whether PLZF protein can bind to the promoters of SREBP-1c gene and regulate the expression of this gene, we cloned and fused the promoter fragment of SREBP-1c gene (500 bp) to a luciferase reporter gene (pSREBP-1c-500). Also, a number of luciferase reporter constructs were generated that had shorter fragments of the promoter of SREBP-1c gene (pSREBP-1c-224 and p-SREBP-1c-119, p-SREBP-1c-71). The data of the luciferase reporter gene assay revealed that PLZF overexpression enhanced the pSREBP-1c-500, pSREBP-1c-224 and p-SREBP-1c-119 reporter gene transcription in HepG2 cells. Nonetheless, the stimulatory effects of PLZF were eliminated following further truncation of the promoter region to −71 bp (p-SREBP-1c-71) (Figure 3D), which suggests that the sequence between −71 and −119 bp is the mediator for inducing the effects of PLZF on the transcription of SREBP-1c gene. In the next step, using ChIP assays, we examined if endogenous PLZF protein was able to bind directly to the SREBP-1c promoter *in vivo*. Our results indicated that the promoter fragment of SREBP-1c (from +85 bp to −83 bp), which containing the E-box could be amplified while precipitating the DNA complex with anti-PLZF antibody treatment, but not when using normal mouse IgG (negative control) in the liver tissue lysate from C57BL/6J mice (Figure 3E). Finally, mutation of E-box (from CCATGTGC to CaAaaTGC) almost abolished the PLZF regulatory effects (Figure 3D). These data suggested that the effects of PLZF on SREBP-1c gene expression are mediated by the E-box element. These findings indicate that PLZF enhances the transcription of SREBP-1c *via* directly binding to the promoter fragment of the gene.

### Hepatic PLZF increases lipogenesis dependent on interaction with SIRT1

**FIGURE 4:**
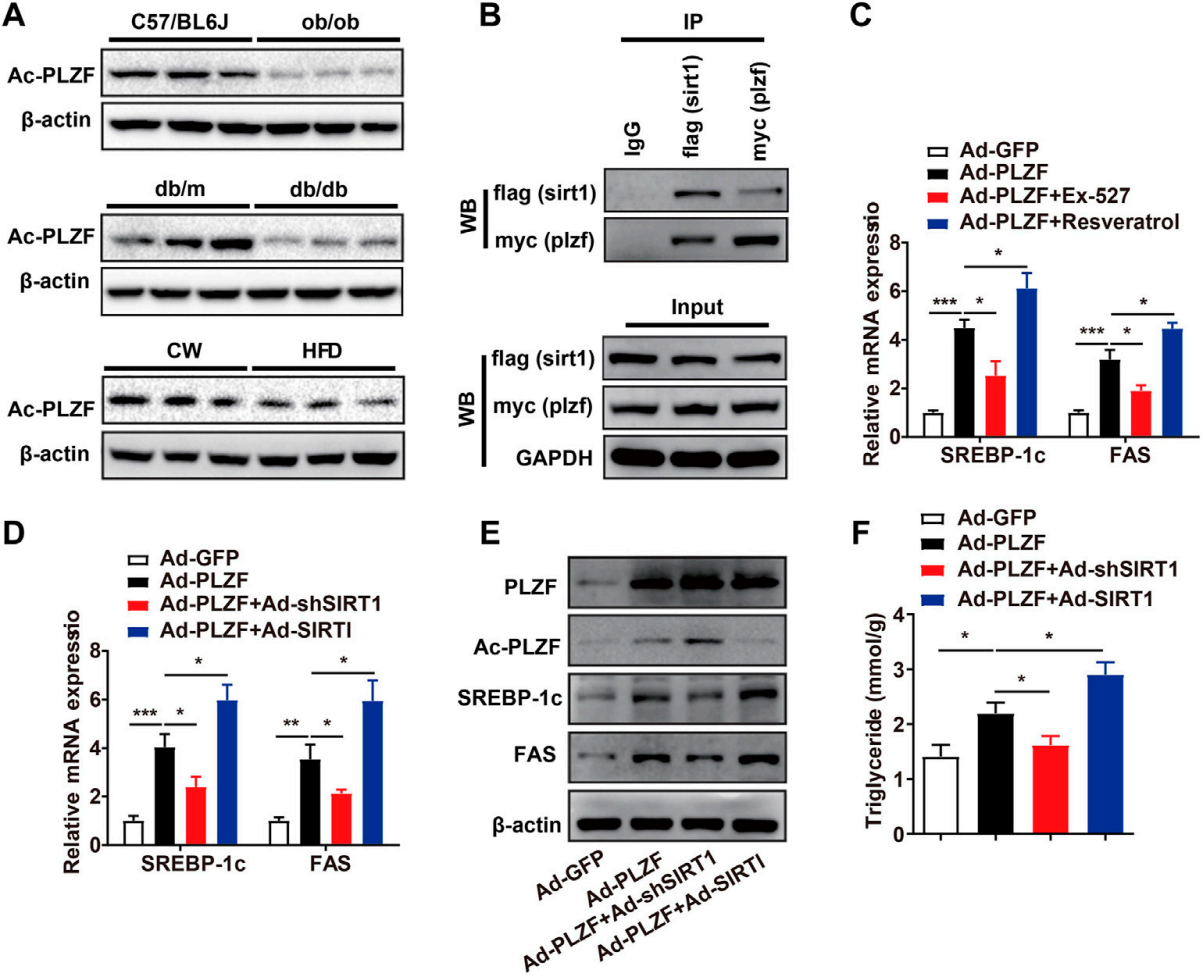
Hepatic PLZF increases lipogenesis, which is dependent on interaction with SIRT1. **(A)** The acetylation level of PLZF in the ob/ob, db/db, and DIO mice, and corresponding littermate controls. **(B)** Co-IP assay showing that PLZF interacts with SIRT1 in the primary hepatocytes. **(C,D)** qPCR analysis of lipogenic genes, including SREBP-1C and Fas with indicated treatment in the primary hepatocytes. **(E)** Western blot analysis of acetylation level of PLZF as well as expression of lipogenic genes in the primary hepatocytes with indicated treatment. **(F)** TG content in the primary hepatocytes treated with indicated treatment. N = 4–6/group. The data shown are the means ± SEM. *p < 0.05, **p < 0.01, ***p < 0.001. EX-527, SIRT1 inhibitor. Resveratrol, SIRT1 activator.

Previous study has revealed PLZF activates interferon-stimulated genes and facilitates natural killer cell functions. The authors’ mechanistic investigation suggests that interferon-induced PLZF phosphorylation and histone deacetylase 1 (HDAC1) recruitment probably mediates the repressor-to-activator conversion (Xu et al., 2009). SIRT1 was identified as an energy sensor and characterized as deacetylates proteins, therefore, we hypothesized that hepatic PLZF-induced dysfunction of lipid metabolism might be mediated by SIRT1 dependent on its deacetylation. As expected, the acetylation level of PLZF was dramatically lowered in the ob/ob, db/db, and DIO mice in comparison to the littermate controls (Figure 4A). Furthermore, our co-immunoprecipitation study on primary hepatocytes, which was incubated with 75 μmol/L palmitic acid for 24 h to mimic the high-fat stress to establish an *in vitro* model of lipid accumulation, revealed that there is a remarkable interaction between PLZF and SIRT1 (Figure 4B). Consistent with our above-mentioned results, silencing of SIRT1 with EX-527, an inhibitor of SIRT1, significantly blocked the PLZF-induced upregulation of lipogenic genes, including SREBP-1c and Fas, while resveratrol treatment, an activator of SIRT1, impeded the PLZF-induced imbalance of lipogenesis (Figure 4C). This phenomenon was also detected in the primary hepatocytes that were Ad-shSIRT1-or Ad-SIRT1-adenovirus-infected (Figure 4D). Notably, SIRT1 knockdown obviously increased the acetylation level of PLZF, which was reduced when Ad-SIRT1 adenovirus was used to overexpress SIRT1 (Figure 4E). Additionally, the PLZF-induced output of TG was also reduced by SIRT1 knockout and was increased by SIRT1 overexpression (Figure 4F).

### Hepatic SIRT1 knockout impedes PLZF-induced hepatic steatosis

**FIGURE 5:**
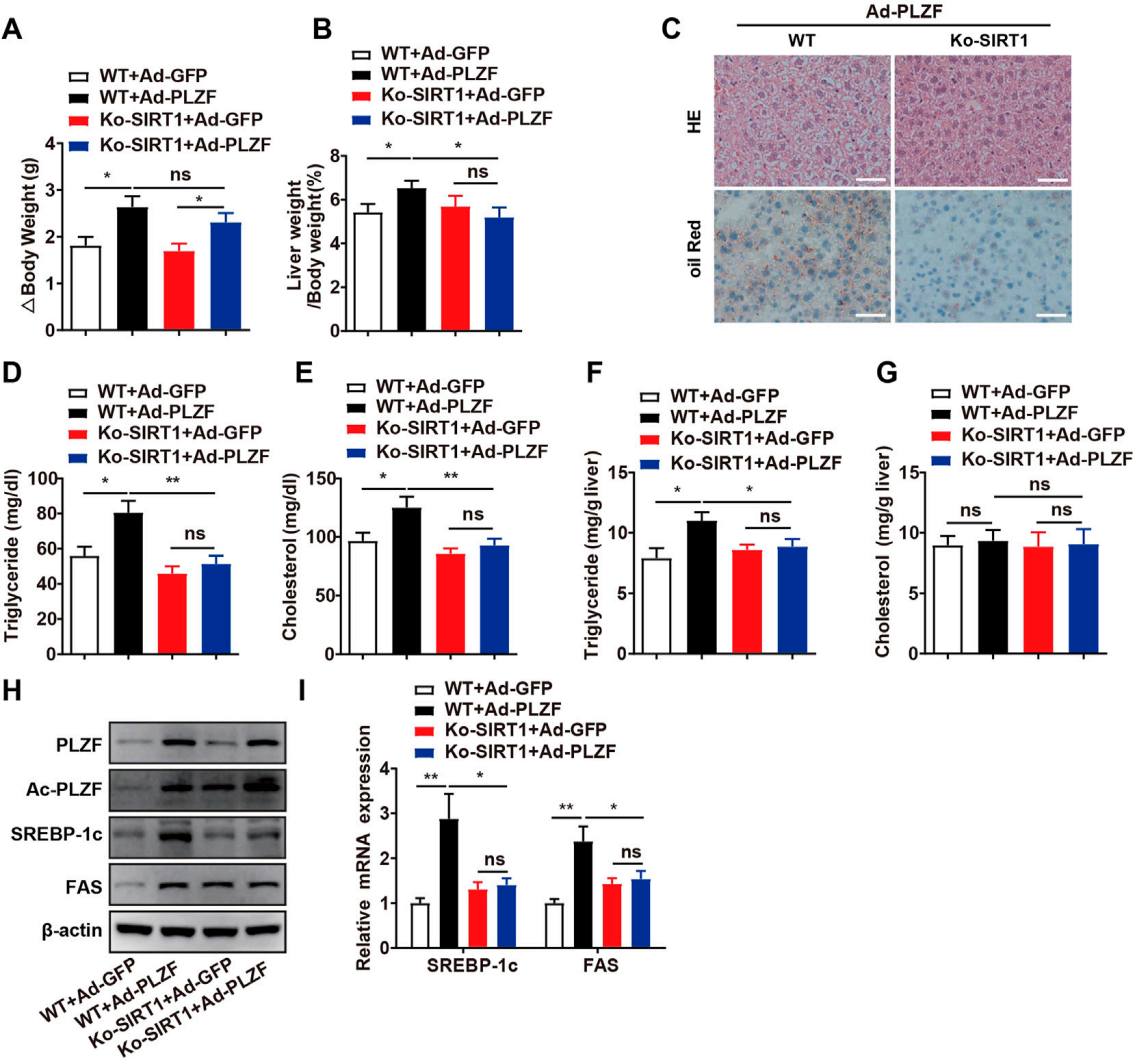
Hepatic SIRT1 knockout impedes PLZF-induced hepatic steatosis. Male C57BL/6J mice or Ko-SIRT1 mice were injected with Ad-GFP or Ad-PLZF adenovirus and then were sacrificed for further analysis. Depicted are **(A)** body weight change, **(B)** liver weight to body weight ratio, liver sections stained with **(C)** H and E (top panel) and Oil Red O (bottom panel), **(D)** serum TG, **(E)** serum cholesterol, **(F)** hepatic TG, **(G)** hepatic cholesterol, **(H)** acetylation levels of PLZF. **(I)** SREBP-1c and Fas in livers of mice that were treated with the indicated adenovirus were analyzed by qPCR. N = 6–8/group. For adenovirus injection, mice were injected *via* their tail vein with adenovirus. Then, 5–7 days after infection, mice livers and plasma were collected for further analysis. The data shown are the means ± SEM. Scale bar, 50 μm *p < 0.05, **p < 0.01.

Previous research reports have described that overexpression of hepatic SIRT1 induces protection against the metabolic impairment induced through a high-fat diet in mice (Pfluger et al., 2008; Purushotham et al., 2009). Nonetheless, in the basal state, the role of SIRT1 on hepatic lipid metabolism remains poorly understood. Based on co-immunoprecipitation results, SIRT1 interacts with PLZF in a cell-autonomous manner, which will change it from a transcriptional repressor into a transcriptional activator depending on its deacetylation. Therefore, we tested the requirements of SIRT1 in PLZF-induced hepatic steatosis *in vivo*. According to our results, although the hepatic SIRT1 interruption did not diminish the increase in the body weight induced by overexpression of PLZF in the liver, the PLZF-induced increase in the ratio of liver weight to body weight in hepatic-SIRT1-deleted mice was significantly decreased (Figures 5A,B). Moreover, the H&E and Oil Red O stainings results indicated that hepatic SIRT1 knockout partly, but significantly, rescued the apparent hepatosteatosis induced by PLZF overexpression, as was reflected in the decrease of lipid droplets and ballooning degeneration of liver cells (Figure 5C). In contrast to the PLZF-overexpression-induced increase in the intracellular hepatic TG, serum TG and serum cholesterol levels contents of WT mice, in hepatic SIRT1 knockdown mice, PLZF-overexpression induced a partly, but significant, abrogation in the increase of intracellular hepatic TG, serum TG and serum cholesterol contents (Figures 5D–G). Consistent with the attenuation of hepatic steatosis phenotype, our *in vitro* assays further demonstrated a slight, but significant, decrease in deacetylation levels of PLZF in hepatic SIRT1 knockout mice (Figure 5H). Moreover, compared to the littermate controls, the PLZF overexpression induced upregulation of lipogenic genes, including SREBP-1c and Fas, which were obviously abolished by SIRT1 knockout in the liver (Figure 5I).

### Hepatic PLZF overexpression impaired glucose tolerance and insulin sensitivity dependent on SIRT1 deacetylases

**FIGURE 6:**
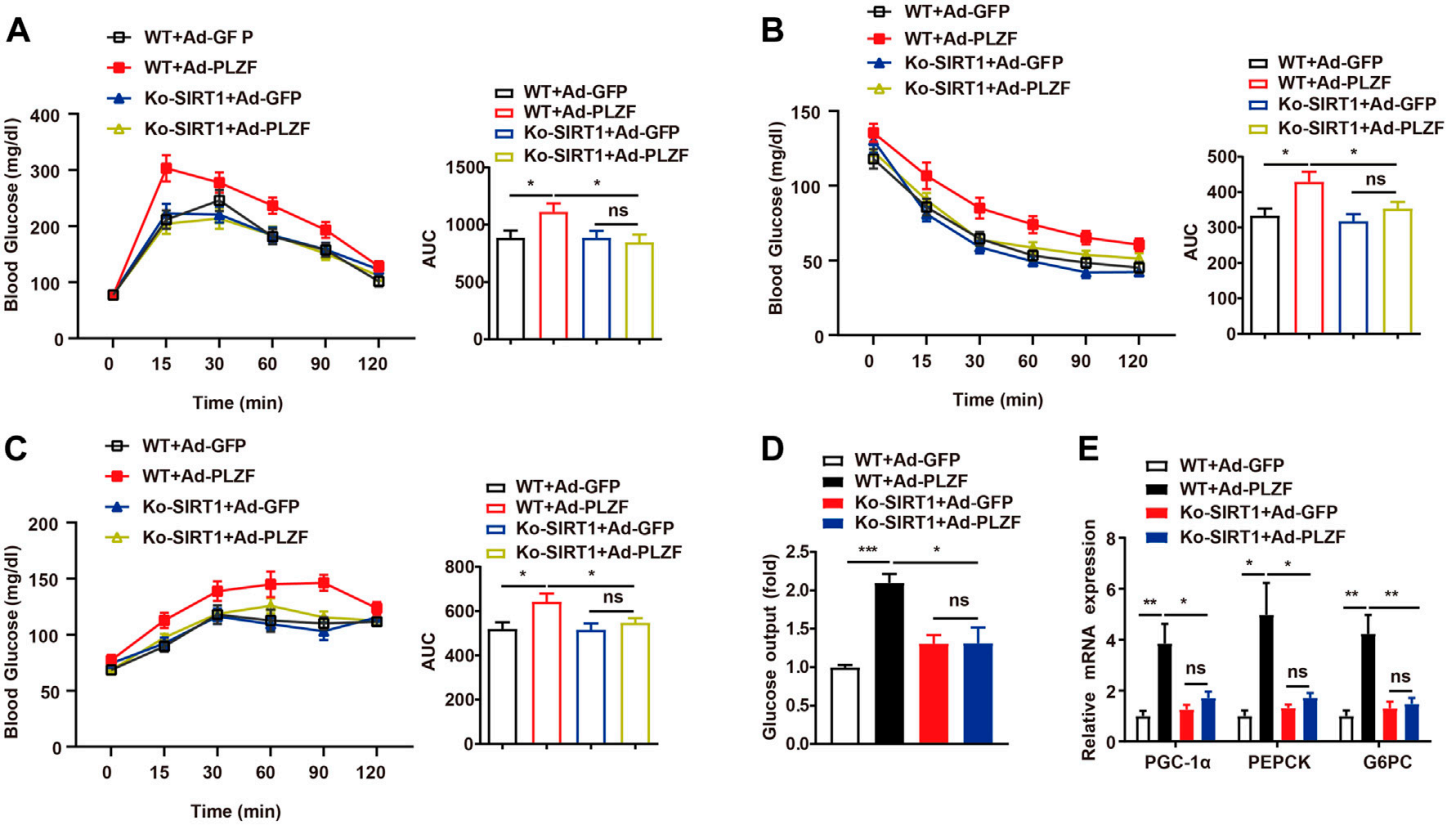
Hepatic PLZF overexpression impaired glucose tolerance and insulin sensitivity, which was dependent on SIRT1 deacetylases. Ad-GFP or Ad-PLZF adenovirus was injected into C57BL/6J mice (male) or Ko-SIRT1 mice. Blood glucose levels during **(A)** GTT, **(B)** ITT, and **(C)** PTT in mice that were infected with the indicated adenovirus. **(D)** Glucose production of the primary hepatocytes isolated from male C57BL/6J mice or Ko-SIRT1 mice infected with the indicated adenovirus. **(E)** For livers of mice infected with the indicated adenovirus, PGC-1α, PEPCK, and G6PC were analyzed using qPCR. N = 6–8/group. For adenovirus injection, mice were injected *via* their tail vein with adenovirus. Then, 5–7 days after infection, mice livers and plasma were collected for further analysis. The data shown are the means ± SEM. *p < 0.05, **p < 0.01.

A previous report has demonstrated a pivotal role for PLZF in the homeostasis of the metabolism of glucose by the positive regulation of gluconeogenesis and negative effects on the insulin signaling pathway (Chen et al., 2014). To further investigate whether SIRT1 was involved in PLZF-induced impairments of systemic glucose/insulin sensitivity, we carried out the glucose-tolerant test (GTT) and insulin-tolerant test (ITT) in SIRT1^−/−^ mice with hepatic overexpression of PLZF. Consistent with this previous report, in our study, mice on a standard diet with PLZF overexpression in the liver showed a clear impairment of glucose tolerance and insulin sensitivity (Figures 6A,B). Interestingly, when SIRT1 was knocked down in the liver, the dysfunction of systemic glucose tolerance and insulin sensitivity induced by overexpression of hepatic PLZF appeared to be partly rescued (Figures 6A,B). Meanwhile, SIRT1 knockout in the liver also alters PLZF-induced disorder of hepatic glucose production, which was illustrated by the results of the pyruvate tolerance test (PTT) (Figure 6C). To further examine the underlying mechanism in place, we examined the task of SIRT1 loss-of-function in the PLZF-regulated change of gluconeogenic genes. Our results showed that the increase of glucose production from primary hepatocytes with PLZF overexpression was obviously rescued by SIRT1 knockout (Figure 6D). In addition, the upregulation of gluconeogenic genes, including PGC-1α, PEPCK, and G6PC that are mediated by the overexpression of hepatic PLZF, was reversed by disruption of SIRT1 in the liver (Figure 6E). These data suggested that hepatic PLZF manipulated lipid and glucose metabolism that is dependent on SIRT1 deacetylase.

### Hepatic PLZF overexpression induces the expression of inflammatory factors and inhibits mitochondrial biogenesis *via* SIRT1

**FIGURE 7:**
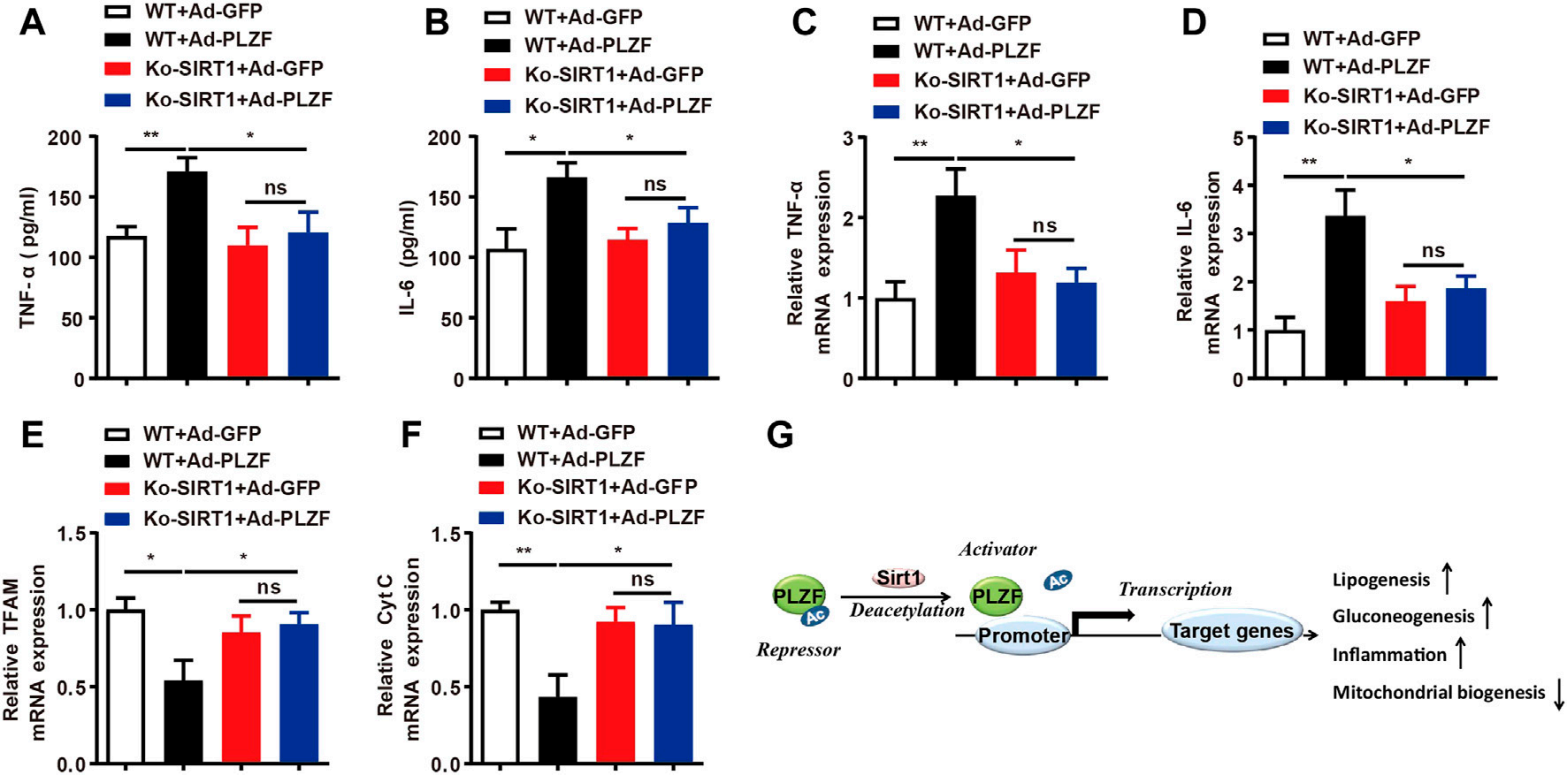
Hepatic PLZF overexpression induces the expression of inflammatory factors and inhibits mitochondrial biogenesis through SIRT1. **(A,B)** Serum TNFα and IL-6 levels were determined with ELISA assay in the mice with indicated treatment. **(C,D)** Liver TNFα and IL-6 mRNA expression was determined with qPCR assay in the mice with indicated treatment. **(E,F)** Liver TFAM and Cyt C mRNA expression was determined with qPCR assay in the mice with indicated treatment. **(G)** Schematic representation of PLZF mediated hepatic lipid homeostasis. N = 6–8/group. For adenovirus injection, mice were injected *via* their tail vein with adenovirus. Then, 5–7 days after infection, mice livers and plasma were collected for further analysis. The data shown are the means ± SEM. *p < 0.05, **p < 0.01.

Inflammatory processes play a crucial role in the pathogeneses of fatty liver diseases, where constant inflammation contributes to the further advancement of hepatic steatosis (Sutti and Albano, 2020). Taking into account that hepatic PLZF overexpression induces hepatic steatosis and PLZF plays a crucial role in the natural killer cell function, we hypothesized that overexpression of PLZF will increase the contents of intrahepatic pro-inflammatory cytokine TNFα and IL-6. Indeed, hepatic PLZF overexpression significantly increased the serum TNFα and IL-6 levels (Figures 7A,B), and their mRNA expression in the liver was also dramatically promoted (Figures 7C,D). In contrast, the upregulation of TNFα and IL-6 induced by the overexpression of PLZF was blocked by SIRT1 disruption (Figures 7A–D). Many studies have been indicative of an association between hepatic steatosis and the mitochondrial dysfunction (Sunny et al., 2017). Therefore, we also decided to examine how PLZF overexpression regulates the function of mitochondria and whether SIRT1 mediates this regulation. Interestingly, the expression of mitochondrial transcription factor A (TFAM) and cytochrome c (Cyt C) was decreased in the liver with PLZF overexpression (Figures 7E,F). However, the PLZF-induced mitochondrial dysfunction was eliminated by SIRT1 knockdown (Figures 7E,F).

## Discussion

Herein, we showed that PLZF expression was upregulated in mouse models. C57BL/6J mice with PLZF-overexpressed livers displayed fatty liver phenotype, while the knockdown of hepatic PLZF in db/db and DIO mice alleviated the hepatic steatosis. Molecular mechanisms studies indicated that PLZF activates SREBP-1c gene transcription through binding directly to the promoter fragment of this gene, which would induce a repressor-to-activator conversion depending on its interaction with SIRT1 in the role played by PLZF in the transcription process through deacetylation.

PLZF is recognized as a repressor of transcription *via* the mechanism of recruiting nuclear receptor corepressors one and 2 (NCoR1 and NCoR2) and HDACs for achieving repression; it also takes part in regulating a large number of target genes *via* zinc-finger-recognizable promoter elements (Li et al., 1997; Grignani et al., 1998; Lin et al., 1998; Chauchereau et al., 2004; Guidez et al., 2005). However, in our study, we found that PLZF could be a transcriptional activator instead of a transcriptional repressor, depending on SIRT1-mediated deacetylation. This is in accordance with a recent study, which reported on the PLZF-activation of genes that are stimulated by interferon and the PLZF-promotion of the functions of natural killer cells (Xu et al., 2009). This study clearly showed that upon stimulation of PLZF by IFN, PLZF would act as an activator of transcription instead of a repressor of it, as was recognized beforehand. The mechanistic analysis of this study indicated that IFN enhanced phosphorylation-dependent binding of PLZF to HDAC1 to mediate the conversion of repressor-to-activator. The phosphorylation of PLZF was performed within the BTB domain, probably *via* the c-Jun amino-terminal kinase (JNK) cascades. This phosphorylation was required for interferon-stimulated gene induction. Considering there is no commercial anti-phospho PLZF antibody is available at present, we have not detected the phosphorylation level of PLZF. However, in the future, further examination of the level of phosphorylated PLZF in the primary hepatocytes or tissues derived from mouse model of NAFLD could be detected by immunoprecipitated with a PLZF antibody firstly and then immunoblotted with anti-phospho Ser, anti-phospho Tyr antibodies. In addition, it was found that cyclin-dependent kinase CDK2 induced phosphorylation of another domain of PLZF, which resulted in impairment of the repression of the transcription; this indicated that phosphorylation could be counteracted by repression (Costoya et al., 2008). Interestingly, another study revealed that following IFN stimulation, the phosphorylated STAT1 (STAT = signal transducers and activators of transcription) was acetylated by one HAT, namely CREB binding protein (CBP) (Krämer et al., 2009). The acetylated STAT1 was then sequestered in the cytoplasm, and could no longer stimulate transcription. Therefore, HDACs induce deacetylation of STAT1 and restore its transcriptionally activated state. Thus, the need for HDAC in the transcription that is stimulated by INF may not be to the degree of chromatin, but might be representative of an acetylation-to-deacetylation switch that finely regulates the function of an activator important for interferon-stimulated genes transcription. The reciprocal dynamic action of HATs and HDACs was suggested to account for the need for HDAC activity for the functioning of other genes. Another finding that provides support to a dynamic exchange of acetylation-deacetylation between HDACs and HATs is that some HDACs and HATs have been found to be in the near vicinity of each other (Yamagoe et al., 2003). The HAT-HDAC dynamics-regulated STAT1 acetylation is interesting because the acetylation of PLZF is also carried out by p300, which is a HAT with close connections to CBP (Guidez et al., 2005). Also, this acetylation has been found to be a requirement for the transcriptional repressor activity of PLZF. Thus, we propose that the state of PLZF transcription might be characterized by a dynamic switching between acetylation and phosphorylation, because IFN stimulation induces the phosphorylation of PLZF, similar to STAT1. Formation of these different transcriptional complexes may be regulated by complicated post-transcriptional modifications of PLZF. Further studies are necessary for elucidating the double character of PLZF as a transcriptional repressor and a transcriptional activator and the dynamic post-translational modifications in place (Ozato, 2009).

Previous studies have demonstrated that among lymphoid cells, PLZF expression is highly restricted to iNKT cells and has not been detected in B cells and NK cells. PLZF was also not detected in eosinophils, neutrophils, or macrophages (Kovalovsky et al., 2008; Fu et al., 2020), which was consistent with the data in BioGPS database (http://biogps.org/#goto=genereport&id=235320). In addition, with qPCR and western blotting analysis, we conformed that PLZF was relatively high expressed in liver and adipose tissues (Supplementary Figure S3). PLZF is a transcription factor specific to iNKT cells, which is required for the full functionality of such cells (Kovalovsky et al., 2008). In models of NAFLD, activation of the immune system played a role in the further progress of damage caused by the fatty liver, where iNKT cells were found to be sensitive to lipid antigens and could show cytotoxicity against hepatocytes (Crosby and Kronenberg, 2018). It is possible to induce NASH-like liver pathology using diets that are deficient in choline, or methionine choline or contain high amounts of fat and cholesterol; this would result in the elevated expression levels of CD1d, accumulation of intrahepatic iNKT cells and consequently, enhanced fibrinogenesis, higher levels of alanine aminotransferase (ALT) and increased NASH disease scores (Syn et al., 2010; Syn et al., 2012; Wolf et al., 2014; Bhattacharjee et al., 2017). In addition, activation of iNKT cells by lipid excess contributes to inflammation of the tissue, resistance toward insulin, and hepatic steatosis in obese mice (Wu et al., 2012). All these data indicated that PLZF may control hepatic metabolism through iNKT cells by producing paracrine mediators.

SNP (783C>G) in the PLZF coding sequence results in nonsynonymous amino acid substitution–serine to threonine at position 208 (T208S)–which affects total body weight, adiposity, and the insulin sensitivity of skeletal muscle (Seda et al., 2005). PLZF is an element of a transcription factor family that is a carrier of the Pox virus and Zinc finger-Bric-a-brac Tramtrack Broad complex (POZ-BTB) domain and Kruppel type C2H2 zinc fingers. PLZF takes part in the regulation of numerous target genes *via* zinc-fingers-recognizable promoter elements. The protein region flanking the substitution containing POZ-BTB domain and C2H2 zinc fingers may be affected by the SNP. Therefore, the target genes regulating hepatic steatosis and related metabolic disorders could not be recognized and transcripted by the zinc fingers. However, further studies utilizing PLZF containing this SNP are necessary to determine whether this minor difference is metabolically “active” or “silent” and reveal the possible underlying mechanisms.

Hyperglycemia is the most effective predictor of hepatic steatosis, where a hyperglycemia-stimulating diet has been found to induce liver steatosis in sheep (Sanders et al., 2018; Kalyesubula et al., 2020). The excess glucose levels not only fuel hepatic *de novo* lipogenesis (DNL) due to being a carbon source for both fatty acids and glycerol synthesis, but also signally prompt upregulation of genes responsible for regulating fatty acid synthesis. The latter could be induced *via* direct and indirect processes: 1) in the direct scenario, the carbohydrate response element binding protein (chREBP) is essential (Postic et al., 2007), and 2) in the indirect one, insulin-mediated activation of sterol regulatory element binding protein 1c (SREBP-1c) is an important element (Horton et al., 2002). ChREBP is a transcription factor tasked with the regulation of lipogenesis that is stimulated *via* mediation by carbohydrates (glucoses). The activation of ChREBP by glucose is *via* the regulation of the entry of ChREBP into the nucleus and by inducing the activation of transcription factor-DNA binding. Stimulation by glucose is required for binding of ChREBP to the promoter of liver-type pyruvate kinase (L-PK), which is a crucial enzyme that takes part in the regulation of glycolysis. The L-PK catalyzes phosphoenolpyruvate-to-pyruvate conversion, which steps into Krebs cycle to produce citrate, which is another source of acetyl-CoA utilized for the synthesis of fatty acids (Stoeckman et al., 2004; Ahmed and Byrne, 2007). Srebf1 encodes SREBP-1c, a key transcription factor that is tasked with the regulation of the biosynthesis of lipids by putting under control the expression of various different enzymes essential for endogenous cholesterol, fatty acid, triacylglycerol, and phospholipid synthesis (Eberlé et al., 2004; Fu et al., 2020). Herein, we report that PLZF regulates the biosynthesis of lipids by binding to the SREBP-1c promoter region that regulates the expression of SREBP-1c. Interestingly, a recent report revealed that PLZF contributed to the expression of gluconeogenic genes and hepatic glucose output, resulting in hyperglycemia (Chen et al., 2014). Thus, all these data indicated that PLZF regulates both glucose and lipid production. It is worth noting that our results cannot rule out the possibility that PLZF may induce lipid synthesis, which was partially mediated by hyperglycemia; thus, this issue warrants further investigation in future studies.

In the present study, by means of luciferase reporter gene assay and ChIP analysis, we found that the sequence between +85 and −83 bp that contains the E-box element mediates the effects of PLZF on SREBP-1c gene transcription. In addition, mutation of E-box (from CCATGTGC to CaAaaTGC) almost abolished the PLZF regulatory effects. Thus, such data indicated that the effects of PLZF on SREBP-1c gene expression are through mediation by the E-box element. These findings suggest that PLZF promotes the transcription of SREBP-1c *via* directly binding to its promoter region. However, due to the dual-luciferase reporter assay was conducted in HepG2 cells which are cancer cells and might not really reflect the physiological activity of PLZF on SREBP-1c, therefore, a “normal” hepatocyte cell line, such as AML12 cell, maybe used to confirm this data in future. Furthermore, many studies have revealed the presence of an E-box in the promoter region of both PPARγ (Lee and Ge, 2014; Deng et al., 2016) and FAS gene (Latasa et al., 2000; Jeon et al., 2012; Li et al., 2015). However, whether PLZF directly regulates PPARϒ and FAS gene expression *via* binding to E-box elements needs to be further studied, as the neighboring structure of the E-box element may also be important for effective gene transactivation, our results cannot rule out this possibility. In a recent study, Fu et al. (2020) demonstrated that PLZF and PPARγ2 synergically promotes SREBP-1c transcription to increase lipid biosynthesis in iNKT cells. However, the synergism of PPARγ2 and PLZF in regulating the SREBP-1c expression was not investigated in our study. In the future, co-immunoprecipitation of PLZF and PPARγ in HFD mice derived liver tissues should be performed to determine the interaction between them. In addition, PLZF and PPARγ2 should be co-overexpressed to further evaluate the SREBP-1c expression in primary hepatocytes.

In conclusion, we report on the identification of hepatic PLZF as a novel candidate gene for NAFLD *via* binding to the promoter of SREBP-1c and upregulating lipogenic genes. Most importantly, we found that the requirement of hepatic SIRT1 for the dynamic switch from being a transcriptional repressor to being a transcriptional activator of PLZF depends on its deacetylase. This may present a potential therapeutic target for future attempts to treat NAFLD.

## Data Availability

The original contributions presented in the study are included in the article/Supplementary Material, further inquiries can be directed to the corresponding authors.
