# Supplementary material for: Mouse promyelocytic leukemia zinc finger protein (PLZF) regulates hepatic lipid and glucose homeostasis dependent on SIRT1
Source: Front Pharmacol. 2022 Nov 10;13:1039726. doi: 10.3389/fphar.2022.1039726 (PMC9684722; doi:10.3389/fphar.2022.1039726)
Supplement: Supplementary file 1 [file Table1.docx]

**Supplementary Materials**

**1 SUPPLEMENTARY DATA**

**Supplementary Figure S1:** (A) In vitro, primary hepatocytes were treated with PA and then qPCR and western blotting were used to to detect the expression of PLZF. (B) Male C57BL/6J mice were injected with Ad-GFP or Ad-PLZF and then accumulated food intake was measured at the indicated times. N = 8/group. The data shown are the means ± SEM.

**Supplementary Figure S2:** Knockdown of hepatic PLZF in DIO mice improves the fatty liver phenotype. DIO mice were injected with Ad-shCON or Ad-shPLZF adenovirus and then were for further analysis. Depicted are (A) body weight change, (B) liver weight to body weight ratio, (C) serum TG, (D) serum cholesterol, (E) hepatic TG, (F) hepatic cholesterol, (G) H&E stained sections (top panel) and Oil Red O staining (bottom panel) of livers, (H) qPCR (left panel) and western blot (right panel) analysis of SREBP-1c, Fas in livers of mice treated with the indicated adenovirus. N = 6-8/group. The data shown are the means ± SEM. ∗p <0.05, ∗∗p <0.01.

**Supplementary Figure S3:** The expression of PLZF in different tissues.

**2 SUPPLEMENTARY FIGURES AND TABLES**

**2.1 FIGURES**

**Supplementary Figure S1**


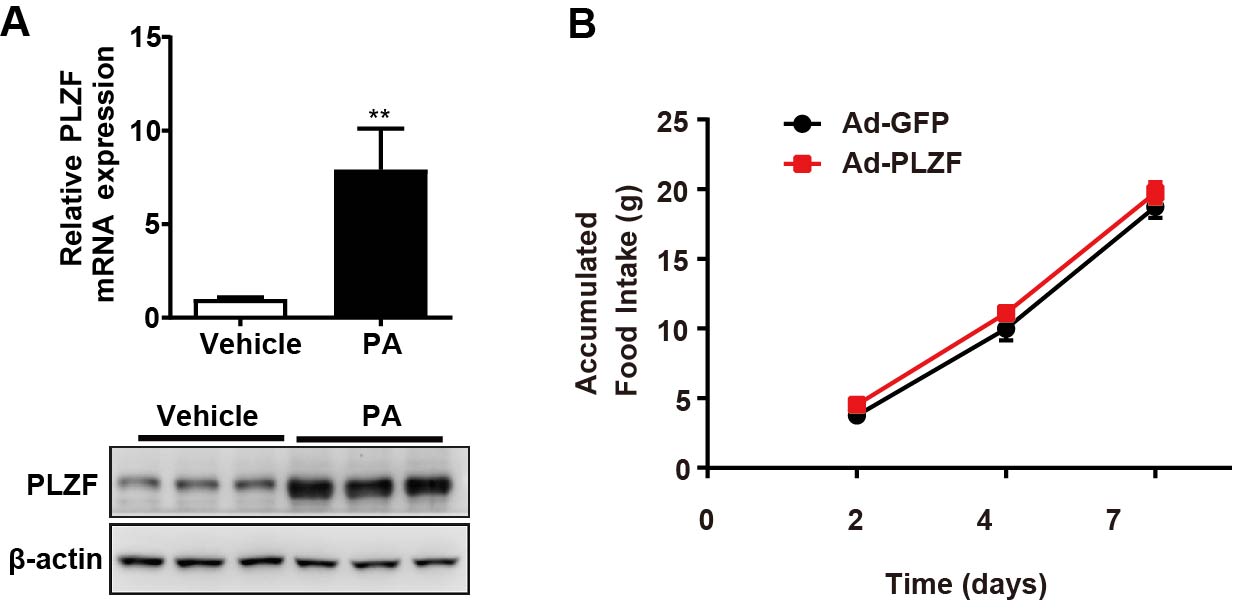


**Supplementary Figure S2**


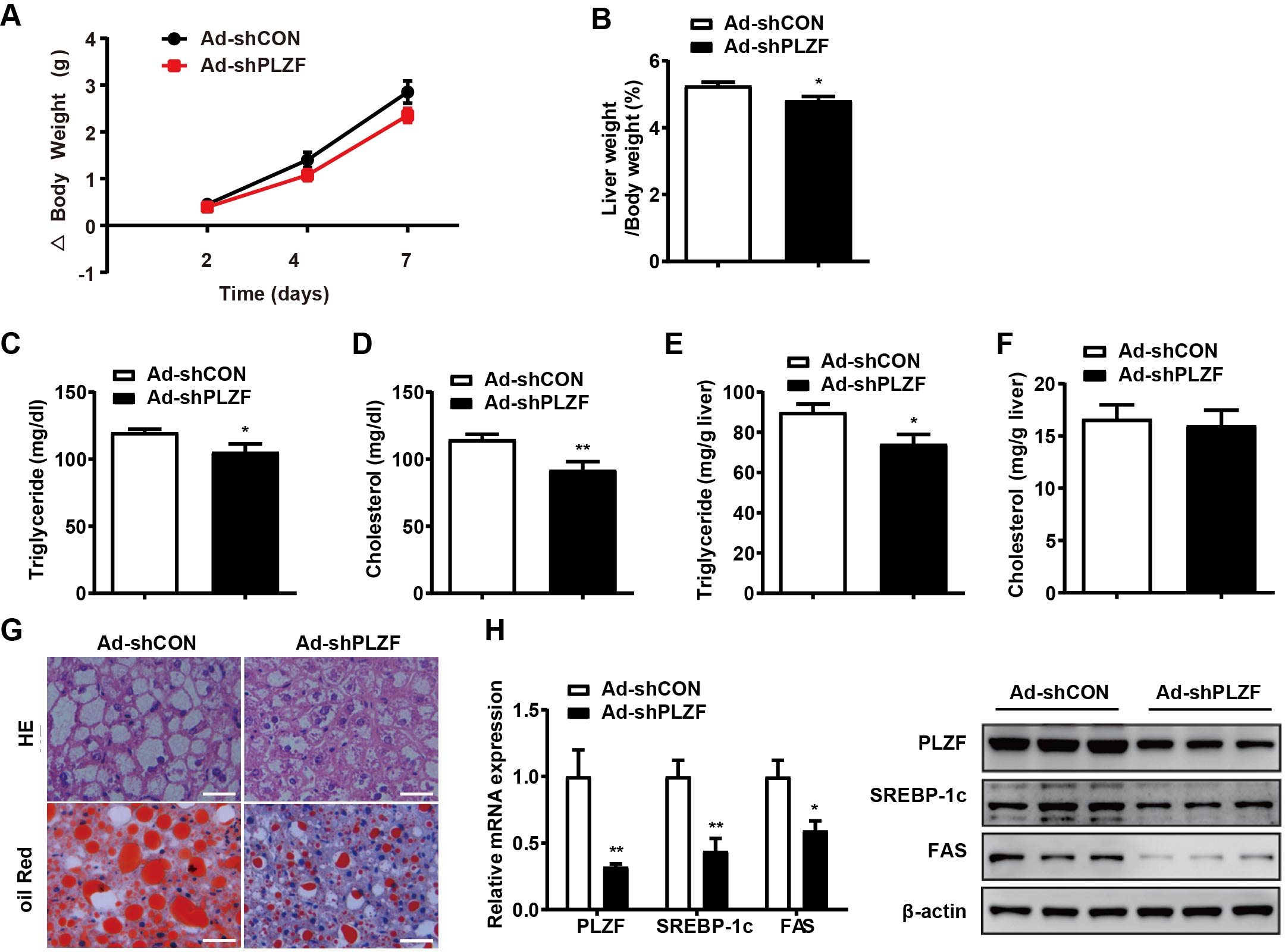


**Supplementary Figure S3**


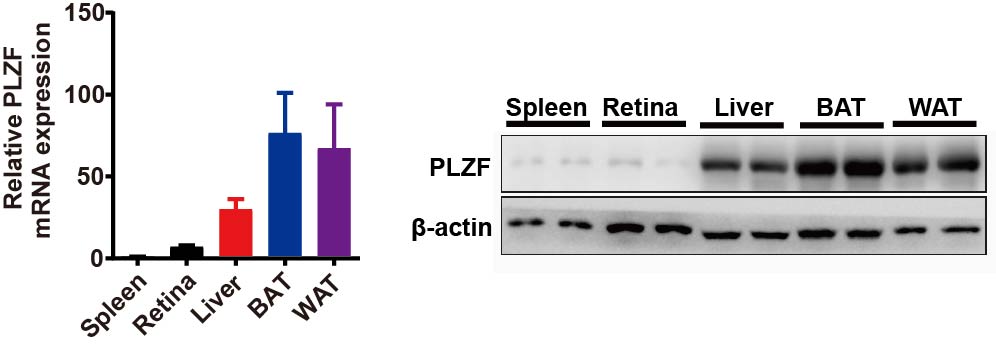


**2.2 TABLES**

Supplementary Table S1. PCR primers used for SREBP-1c promoter amplification

| Gene | Primer sequence (5’-3’) |
| --- | --- |
| p-SREBP-1c-500 | Forward: CCGGGTACCATGACCCTGCACCACCAACT |
|  | Reverse: CCGCTCGAGTTCACCTGTCAGGCCCCGCGA |
| p-SREBP-1c-244 | Forward: CCGGGTACCTAACCCCAGCGCGCGCTGGCGCA |
|  | Reverse: CCGCTCGAGTTCACCTGTCAGGCCCCGCGA |
| p-SREBP-1c-119 | Forward: CCGGGTACCGTGGGCGGGGCCCTAAT |
|  | Reverse: CCGCTCGAGTTCACCTGTCAGGCCCCGCGA |
| p-SREBP-1c-71 | Forward: CCGGGTACC GCGCTCACCCGAGGGGC |
|  | Reverse: CCGCTCGAGTTCACCTGTCAGGCCCCGCGA |

Supplementary Table S2. Gene-Specific Primers Used for RT-PCR Analyses

| Gene | Primer sequence (5’-3’) | Amplicon (bp) |
| --- | --- | --- |
| PLZF | Forward: CTGCGGAAAACGGTTCCTG | 150 |
|  | Reverse: GTGCCAGTATGGGTCTGTCT |  |
| SREBP-1a | Forward: GGCCGAGATGTGCGAACT | 69 |
|  | Reverse: TTGTTGATGAGCTGGAGCATGT |  |
| SREBP-1c | Forward: GGAGCCATGGATTGCACATT | 70 |
|  | Reverse: GGCCCGGGAAGTCACTGT |  |
| FASN | Forward: GGAGGTGGTGATAGCCGGTAT | 140 |
|  | Reverse: TGGGTAATCCATAGAGCCCAG |  |
| LXRα | Forward: CCTTCCTCAAGGACTTCAGTTACA | 98 |
|  | Reverse: CATGGCTCTGGAGAACTCAAAGAT |  |
| PGC-1α | Forward: TGGACGGAAGCAATTTTTCA | 145 |
|  | Reverse: TTACCTGCGCAAGCTTCTCT |  |
| G6PC | Forward: CGACTCGCTATCTCCAAGTGA | 173 |
|  | Reverse: GTTGAACCAGTCTCCGACCA |  |
| PEPCK | Forward: CAGGATCGAAAGCAAGACAGT | 108 |
|  | Reverse: AAGTCCTCTTCCGACATCCAG |  |
| GK | Forward: TGAGCCGGATGCAGAAGGA | 75 |
|  | Reverse: GCAACATCTTTACACTGGCCT |  |
| TNF-α | Forward: AGGGTCTGGGCCATAGAACT | 103 |
|  | Reverse: CCACCACGCTCTTCTGTCTAC |  |
| IL-6 | Forward: AGGAGACTTCACAGAGGATACC | 126 |
|  | Reverse: GAATTGCCATTGCACAACTCTT |  |
| TFAM | Forward: TCGCATCCCCTCGTCTATCA | 118 |
|  | Reverse: TTTGGGTAGCTGTTCTGTGG |  |
| CytC | Forward: TCCATCAGGGTATCCTCTCC | 131 |
|  | Reverse: GGAGGCAAGCATAAGACTGG |  |
| β-actin | Forward: CTCTGGCTCCTAGCACCATGAAGA | 200 |
|  | Reverse: GTAAAACGCAGCTCAGTAACAGTCCG |  |
